# Book and Pamphlet Notices

**Published:** 1860-06

**Authors:** 


### BOOK AND PAMPHLET NOTICES.

Lectures on the Diseases of Infancy and Childhood, by Charles West, M. D., author of "Lectures on the Diseases of Women," &c., &c. Third American edition from the Fourth revised and enlarged London edition. Blanchard & Lea, Philadelphia, 1860. Received through S. C. Griggs & Co., 39 and 41 Lake street.

There are few authors who have added more valuable contributions to medical literature than Dr. West, and the subjects on which he has written are among those of the first importance to the profession. The opinion is not unfrequently met with among the people, that the diseases of infancy and childhood are not capable of being benefited by medical treatment; and even members of the profession are not wanting who avow that such cases should be left to the care of experienced nurses, or trusted to the restorative powers of nature. Than this conclusion none could be more erroneous. The maladies of childhood, when correctly apprehended, are quite as amenable to medical treatment as are those of adult life, and perhaps even more so, on account of the easy susceptibility of the physical organization, at this period of life, to the influences of external circumstances, and for this reason the ravages of disease, if left unimpeded, have, in such cases, the most fatal tendencies. Let this fact be fully appreciated, and then will diseases of childhood come to receive something of the attention that their importance merits. The statistics of the most favored countries show that about one-half of the mortality of the human race occurs prior to the age of five years, and this high ratio of fatality is undoubtedly, to some extent, due to the fact that practitioners fail to recognize the modifications that infancy gives to disease, and to the effects of therapeutical appliances. We hardly think that Dr. West goes too far, when he says that those who would successfully treat the diseases of childhood "have to study a new semiology, to learn a new pathology, and new therapeutics." The author says "These Lectures embody the results of 900 observations, and 228 post mortem examinations made among nearly 30,000 children, who, during the past twenty years, have come under my care."

---

The Peninsular and Independent Medical Journal, published at Detroit, Mich., is discontinued at the end of the second volume, for the alleged reason "that it does not pay." That a publication so favorably located, should be obliged to suspend for such a cause, is a fact by no means cheering to others embarked in the enterprise of Medical Journalism.

